# Knowing is half the battle: Assessments of both student perception and performance are necessary to successfully evaluate curricular transformation

**DOI:** 10.1371/journal.pone.0210030

**Published:** 2019-01-11

**Authors:** Tarren J. Shaw, Suann Yang, Troy R. Nash, Rachel M. Pigg, Jeffrey M. Grim

**Affiliations:** Department of Biology, Presbyterian College, Clinton, South Carolina, United States of America; University of Westminster, UNITED KINGDOM

## Abstract

Student-centered pedagogies increase learning and retention. Quantifying change in both student learning gains and student perception of their experience allows faculty to evaluate curricular transformation more fully. Student buy-in, particularly how much students value and enjoy the active learning process, has been positively associated with engagement in active learning and increased learning gains. We hypothesize that as the frequency of students who have successfully completed the course increases in the student population, current students may be more likely to buy-in to the curriculum because this common experience could create a sense of community. We measured learning gains and attitudes during the transformation of an introductory biology course at a small, liberal arts college using our novel curriculum, Integrating Biology and Inquiry Skills (IBIS). Students perceived substantial learning gains in response to this curriculum, and concept assessments confirmed these gains. Over time, buy-in increased with each successive cohort, as demonstrated by the results of multiple assessment instruments, and students increasingly attributed specific components of the curriculum to their learning. These findings support our hypothesis and should encourage the adoption of curricular transformation using IBIS or other student-centered approaches.

## Introduction

Initiatives such as the Vision and Change in Undergraduate Biology Education [1] and the Royal Society’s State of the Nation reports [2] call for change in undergraduate science education and provide recommendations based on the educational literature, especially adoption of active learning pedagogies (summarized in [3]). In response to these initiatives, we developed a new introductory biology curriculum called Integrating Biology and Inquiry Skills (IBIS). A primary design feature of IBIS is guided, student-centered inquiry [4]. The curriculum promotes critical thinking by utilizing inquiry activities in the lecture session, paired with open-ended, inquiry laboratory investigations. Lecture content is introduced to students via scenarios and case-studies instead of a sequential march through the textbook. Substantial time is devoted to application of knowledge and group discussions of student-generated questions. Both formative and summative assessments were aligned with learning objectives and prioritized critical thinking and application over recall and recognition. Course concepts are further emphasized in the paired laboratory curriculum where student teams work under the guidance of faculty and experienced upper-level students (undergraduate peer mentors) to develop and carry out original experiments over multiple two-week periods. These experiments culminate in a lab report that models the format and style of a scientific article.

Active learning techniques achieve higher learning gains than traditional lecture, particularly for underrepresented minorities in STEM (Science, Technology, Engineering, and Math) [3]. However, students must be engaged as active learners to maximize the impact of active learning techniques [5]. Student buy-in, i.e., how much students value and enjoy the active learning process, can be positively associated with engagement in active learning [5]. Thus, a greater understanding of student buy-in could aid curricular transformation. For example, a barrier to student buy-in may be the novelty of active learning [6]. According to Expectancy Violation Theory, student resistance can occur when class requirements conflict with student expectations based on their previous experiences [6]. If students expect to be passive recipients of information, then any active learning environment will violate their expectations and might cause dissatisfaction. Increasing buy-in can result when students attribute the magnitude of their learning gains to the specific aspects of the active learning environment [5]. We hypothesize that although a new curriculum may not prompt many students in the first cohort to identify that active learning experiences are related to perceived learning gains, each successive cohort should increasingly attribute their learning gains to active learning. As the frequency of students who have successfully completed the course increases in the student population, current students may be more likely to buy-in to the curriculum because this common experience could create a sense of community.

To detect if student buy-in to a new curriculum changes over time, we documented student opinion, self-efficacy, and learning gains in multiple consecutive years while employing the IBIS curriculum. Thus, we not only measured learning gains to evaluate curricular effectiveness, but also examined the role of student opinion and affect (student identity, self-efficacy and sense of community; [7]) with multiple assessment tools to identify student perceptions of new classroom experiences. In each year of the IBIS program, students demonstrated significant learning gains in course outcomes by the conclusion of the course. Further, over the four years of the program, students increasingly recognized that the course structure and pedagogy contributed to their learning gains, and the interest of STEM majors in learning more about biology was consistently maintained. We reflect on these data to make recommendations for institutions looking to promote curricular reform, including the adoption of multiple assessments targeting different facets of the classroom experience to gauge the efficacy of curricular reform in terms of student performance and buy-in.

## Materials and methods

We evaluated the IBIS program at a small, liberal arts college in the US over a four-year period (2013–2016). We assessed student learning gains and students’ evaluations of their own performance. Student learning gains were measured with a pre- and post-course concept assessment, while attitudinal changes and perceptions of learning were assessed with a post-course Student Assessment of Learning Gains (SALG) survey.

We examined our longitudinal surveys of student attitudes toward the IBIS curriculum and biology as a subject of study. We assessed whether learning gains and attitudes changed over time as a signal that the IBIS curriculum was assimilating into the educational experience and culture at this institution. Additionally, we compared student attitudes between STEM and non-STEM students.

Over the life of the program, 724 students were enrolled in the IBIS curriculum, which was taught by 13 faculty members, six of whom taught in the IBIS curriculum in multiple years. The number of students enrolled in the program during one year varied from 136–245. The number of faculty members instructing the course varied from 4–7 each year (Table 1). In addition to faculty members, the IBIS program also uses undergraduate students as peer mentors (PMs). PMs are students that have previously been successful in the course and are available to help students both in and out of normal class times. We offered pedagogical training sessions for faculty members and PMs who were involved in the IBIS program each year. In these training sessions, we emphasized the importance of explaining how different components of the course relate to student learning. Weekly meetings were also held to reflect on classroom experiences and discuss curricular decisions, such as content revisions, assessments, and implementation strategies.

**Table 1:** Faculty and student participation in the IBIS program.

Year	Total Faculty (Percentage with Previous IBIS Experience)	Total Students (Student to Faculty Ratio in Lecture Sections)^a^	STEM Majors / Non-majors
2013	7 (71%)	245 (30.6:1)	40.7% / 59.3%
2014	6 (67%)	182 (30.3:1)	50.4% / 49.6%
2015	6 (67%)	161 (26.8:1)	61.1% / 38.9%
2016	4 (75%)	136 (27.2:1)	78.1% / 21.9%

^a^Student to Faculty ratio in lab sections ranged from 22.4:1 to 23.3:1

### Assessments

The Presbyterian College IRB approved all data collection (PC-201221). Participants were informed of the IBIS curriculum implementation in their course syllabus. During the first lab meeting of the semester, students were provided an informed consent document, which they signed in order to give the PIs permission to collect and store relevant anonymized data from the study. Data were collected from several consecutive classes of incoming first-year college students. Beginning in 2013, we evaluated student performance and student assessment of learning gains to assess course effectiveness and to guide curricular revisions.

Assessment concepts for the pre- and post-course concept survey were selected based upon several criteria: feedback from our faculty instructors, similarity to questions and topics students might encounter on summative exams, and alignment to common topics found in introductory biology courses [8] and the Biology Concept Inventory (www.bioliteracy.colorado.edu). The survey also included attitudinal questions related to course perceptions, interests, mindset, and self-efficacy. Items used to evaluate student attitudes quantitatively utilized a Likert scale in the pre- and post-course concept survey. We unambiguously matched pre- and post-course responses for 542 students (74.9%); the remainder of responses remained unmatched due to a missing pre- or post-course instrument. Our pre- and post-course survey is available as a supplemental file (S1 File).

To examine further the self-efficacy of student learning, we also developed questions for the SALG instrument (www.salgsite.org, Instruments #63255, #67010, #67900, #71380, #72672, #76196). The SALG instruments had questions structured similarly to the attitudinal questions on the concept survey. The first category provided descriptions of course content/competency and asked students to identify the level of learning gain they perceived as a result of the course. The second category of question asked students to report how helpful they found different elements of the course to be (course structure, components, and other resources). Each of the two categories also asked open-ended questions in which students could expand upon their responses to each set of ranking questions. To evaluate student perceptions of the course we focused on a subset of questions from each of the two categories of questions (S2 File).

### Data analysis

As recommended by [9], pre- and post-course performance on the concept survey were compared in a logistic regression with a generalized linear mixed effects model (binomial errors) with test (pre- vs. post-course) as fixed effects, and student identifier as a random effect. The logistic regression was conducted using the *lme4* package [10] in R [11]. Tests of fixed effects were obtained using the *car* package [12]. *Post-hoc* multiple comparisons were conducted using the *emmeans* package [13]. Normalized learning gains [14], were calculated for visualizing the data and to compare our student performance gains to that of other studies. In addition, we constructed network diagrams to visualize the migration of individual attitudes for the same time period (*bipartite* package in R [15]).

For each question on the SALG, we used chi-squared tests to compare across years the frequencies of student responses for the different ranks of how much gain was attributed to a particular learning outcome or how much help was ascribed to a specific component of the curriculum. We used sentiment analysis methods on the open-ended SALG questions to quantify positive *vs*. negative feelings to different aspects of the curriculum as discussed above. We used the R packages *tm* [16, 17] and *RWeka* [18] to format the text for analysis and extract trigrams (three consecutive words from a response; [19]). We analyzed trigrams, rather than shorter or longer sets of consecutive words because trigrams are the smallest set of consecutive words to which sentiments could be assigned (*e*.*g*., [19]). For each detected trigram, we assigned a sentiment of positive, negative, or unknown. Positive vs. negative sentiment frequency for each category was compared across years using chi-square tests of association with Bonferroni corrections for multiple comparisons to discover if student attitudes with regard to a particular aspect of the program differed between years. All text analyses and subsequent statistical tests were conducted in R [11].

## Results

### Student performance indicators

**Fig 1:**
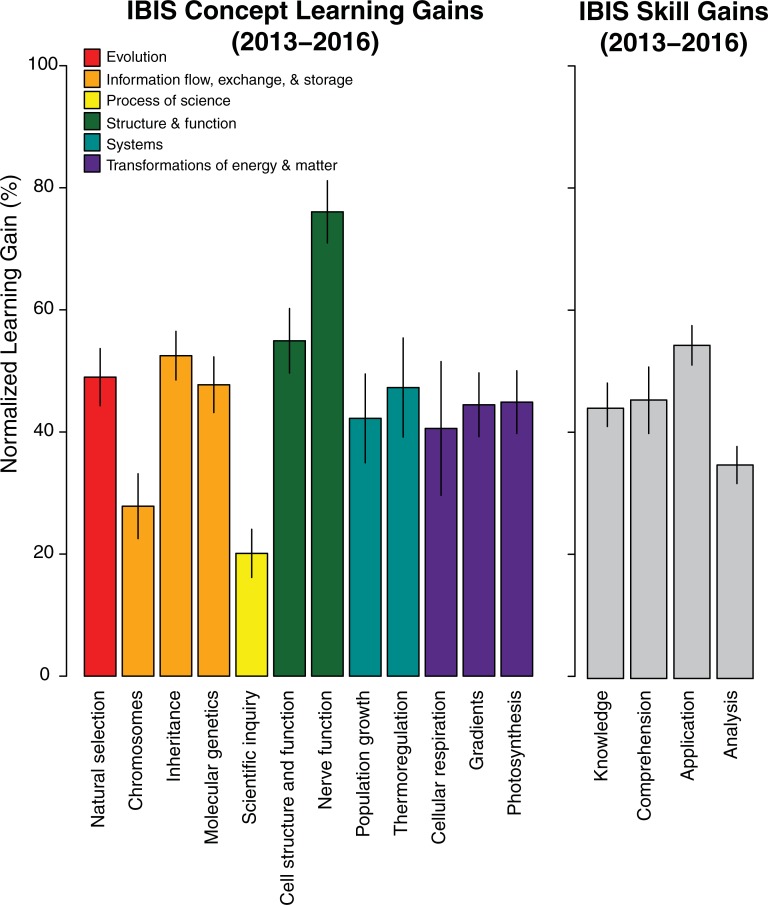
Normalized student learning gains. Normalized learning gains across concepts (a) and skills (b) were above 19%, compiled across all years of the study.

Across all four years, overall course grades did not differ (median grades ranged from 76.2% to 77.1%, *p* = 0.101), however, in each year, students were more likely to answer concept inventory questions correctly after the course (*p* < 0.05 for all years). For concepts, the normalized learning gains were 19–75% each year (Fig 1). For critical thinking skills (knowledge, comprehension, application, analysis [20, 21]), the normalized learning gains were 30–50% for all years (Fig 1).

### Student attitudes

**Fig 2:**
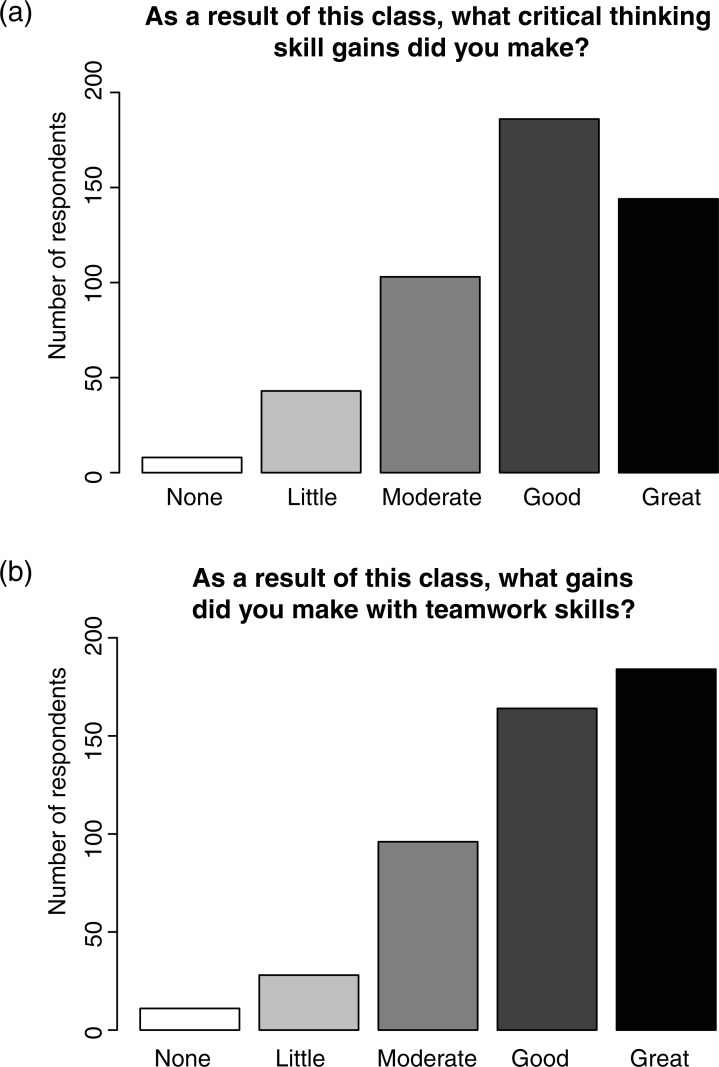
Student perception of learning gains. An overwhelming majority of students, compiled across all years of the study (2013–2016), ranked their gains as moderate or higher in teamwork (a) and critical thinking skills (b) as a result of the course.

**Fig 3:**
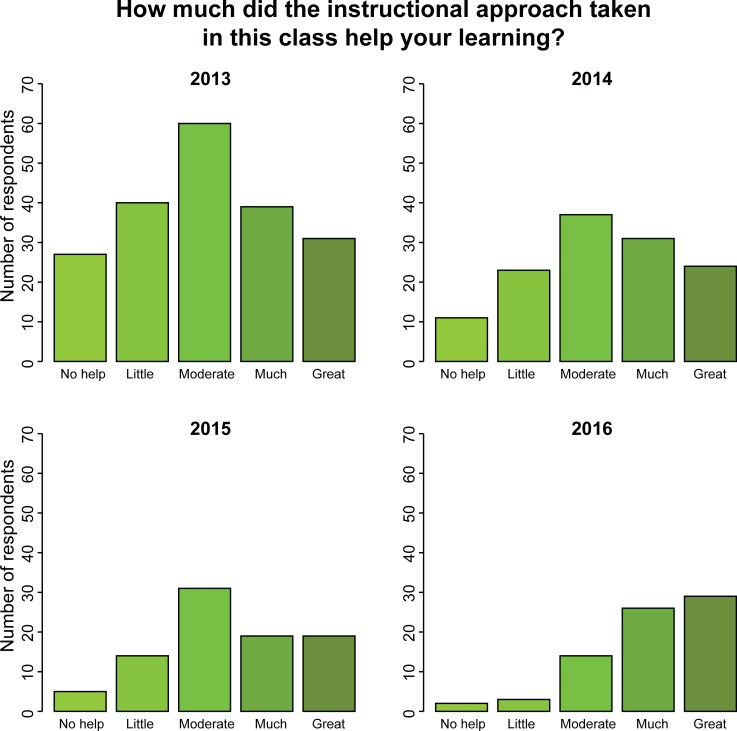
Change in student buy-in over time. Student responses shift over time, reducing the frequency of low rankings (No help, Little) relative to high rankings (Much, Great). Over four years, students increasingly report the instructional methods as responsible for large gains in their learning (*p*<0.0001).

**Fig 4:**
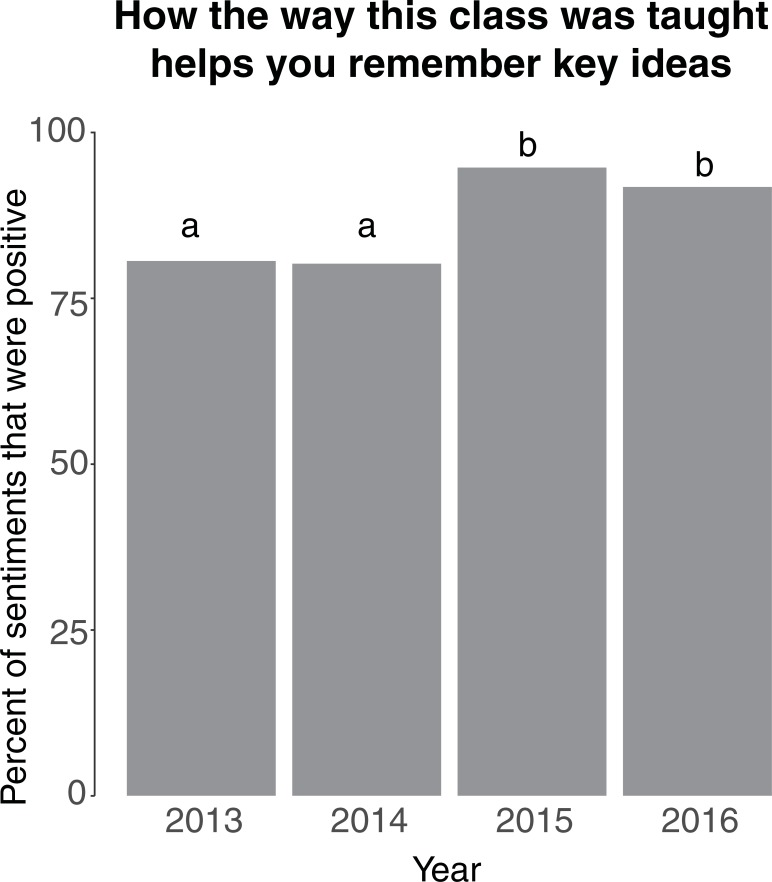
Student sentiment analysis. Regarding pedagogy, positive sentiments were more abundant than negative sentiments in all years, and this proportion was higher in the latter two years (*p*<0.0001). Sentiments were assigned to trigrams (three consecutive words) that were extracted from a free-response survey question.

An overwhelming majority of students perceived their gains as moderate or higher in teamwork and critical thinking skills as a result of the course (compiled across all years; Fig 2). Comparing across years, many aspects of the course–such as the instructional approach, types of formative assessments, and peer mentor roles–were increasingly seen as important for student attitudes toward biology as a discipline and their learning gains (Fig 3, S2 File). When asked to comment on how the course helped them remember key ideas, we found relatively more positive compared to negative trigrams within each year, and more positive trigrams in 2015 and 2016 compared to the first two years of the program (*p*<0.05; Fig 4). “Clicker questions” were most frequently identified as helpful, supporting the findings of [22]. The other most frequent positive trigrams were “helped apply concepts,” “helped retain information,” and “help better understand,” which all suggest that students connected curricular philosophy to their learning gains. Surprisingly, we did not find any difference in how STEM vs. non-STEM majors view active learning pedagogies (S2 File).

**Fig 5:**
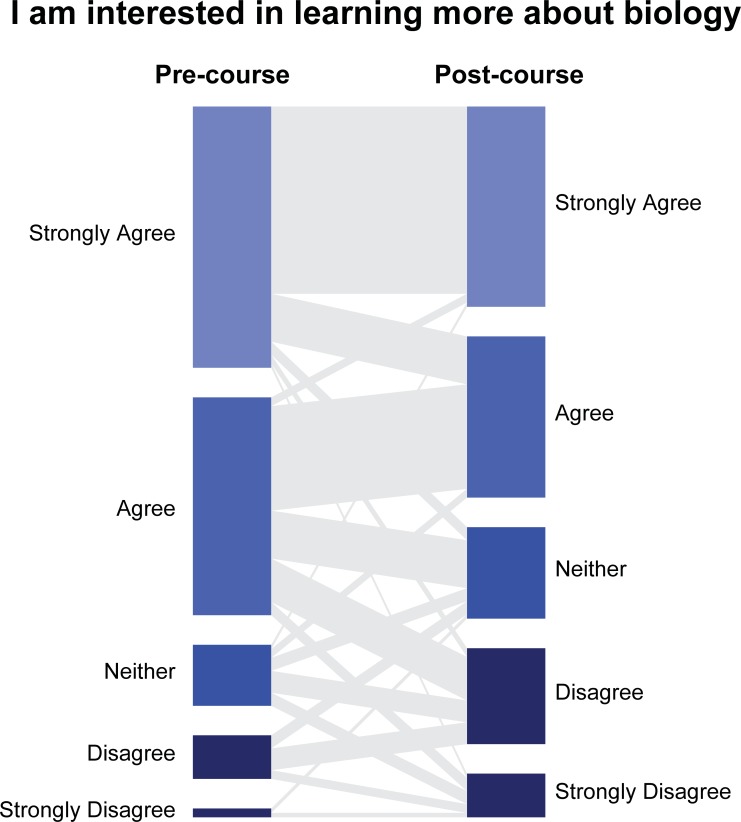
Migration of student attitudes toward biology. Most students maintained a positive view of the subject, though the percentage of students who were uninterested did increase. Students who became less interested were primarily those who were not planning to major in STEM fields.

Within each year, some students became less interested in learning about biology after taking the course (*p* < 0.05 for all years). For example, in 2014, 10% of students either Disagreed or Strongly Disagreed with the statement “I am interested in learning more about biology” prior to taking the course. On the post-course survey, this increased to 24.4% (Fig 5). From 2013 to 2015, most of the disinterest came from students who did not intend to major in STEM fields. Importantly, very few STEM majors reported a reduction in their interest in biology (e.g., in 2014, from 0% Disagree to 3.8% Disagree; no STEM majors Strongly Disagreed). Note that within each year we found relatively more positive (65–81%) compared to negative (35%-19%) trigrams being expressed in response to “How this class changed your attitudes toward biology” (*p*<0.0001). Thus, it appears that although a minority of students became increasingly dissatisfied, the majority were satisfied with the IBIS experience.

## Discussion

We designed and deployed a student-centered introductory biology curriculum at a small, liberal arts college in the US that resulted in both substantial learning gains across all years and increased student buy-in over time. Students participating in the IBIS curriculum demonstrated learning gains between 19–75% (content) and 30–50% (skills), which are comparable to (or exceeding) other reported learning gains from student-centered environments (Fig 1), and help to explain the extensively documented increase in student performance under active learning environments compared to traditional lecturing [3]. For example, [23] measured normalized learning gains between 23% (energetics) and 50% (biological membranes), and [24] reported normalized learning gains between 7–16% (cellular and molecular biology). Normalized learning gains of 48% (cellular respiration) were reported when using a student-centered case study approach [25].

Data collected in parallel on students’ attitudes suggest that students in the IBIS curriculum also assimilate to and recognize the benefits of this instructional curriculum over time (Figs 3 and 4). Thus, it appears that an increased frequency of students who have successfully completed the course in the student population promotes buy-in among current students. In addition, we speculate that the thorough use of formative assessments and a well-structured peer mentor program–major components of the IBIS curriculum–may help students identify and correct deficits in their understanding of course material, which are key components of student metacognition [5]. In addition to helping students during normal class meeting times, PMs created and employed oral quizzes to help students achieve the intended learning outcomes for the course. In other studies, students have indicated that alignment between learning activities and assessments and learning support structures (such as PMs) are important elements in promoting learning [26], and it appears that these elements in the IBIS program also help improve student buy-in (S2 File).

Alternatively, the increase in buy-in to course pedagogy could be associated with the increasing percent of students who aspired to be STEM majors (Table 1). This possibility suggests that aspiring STEM majors could be more receptive to our course pedagogy than non-majors, and student buy-in for majors-only courses may be less difficult to attain. However, we did not find that STEM compared to non-STEM students differed in how much they valued the IBIS instructional approach (**S2 File**). Instead, non-STEM students were more likely to reduce their interest in biology compared to STEM students (Fig 5). Thus, the change in attitudes of non-STEM students could be attributed to a genuine lack of interest in the subject matter and not the way the course was taught.

Students’ perception of and commitment to an active learning environment are thought to be highly correlated to their engagement and success [27]. Although we expected that increased buy-in would be accompanied by increased learning gains [5], we found that learning gains did not change significantly over the course of the study despite improved buy-in. It is possible that the relationship between buy-in and learning gains is more complex than shown previously [5, 27]. Our study underscores the need to evaluate not only learning gains, but also how students perceive their learning environment when assessing curricular transformation.

Emphasizing student buy-in towards curricular and pedagogical changes is important, because student buy-in is crucial to maintain classroom interactions and possibly improve student performance [5, 22, 28]. Instructors should address student expectations of a passive instruction style/classroom early in the semester by sharing the motivation and data behind active-learning instruction. This introduction may help students connect these new experiences to positive impacts on their learning and increase student buy-in [5, 29]. These efforts to promote student buy-in of active learning experiences not only help the perception of an individual faculty’s courses, but also could further reinforce changes in the institutional culture.

## Conclusion

When transforming undergraduate curricula to align with recommended best practices, tracking learning gains is essential; however, student attitudes must also be considered because student buy-in plays a role in creating learning environments that are effective. Thus, instructors and administrators should examine the instruments and methods they use to assess student opinion. Traditional end-of-course evaluations may not adequately assess the student-centered nature of the course or accurately correlate with student learning gains in a course [30], potentially increasing negative perceptions of the curricular changes among students. Our study thus underscores the need for an array of instruments that adequately assess the learning environment’s effect on student performance and perceptions during curricular transformation.

## Supporting information

S1 File Pre- and post-course survey. (PDF)

S2 File Additional SALG analysis. (PDF)
