# Supplementary material for: Knowing is half the battle: Assessments of both student perception and performance are necessary to successfully evaluate curricular transformation
Source: PLoS One. 2019 Jan 11;14(1):e0210030. doi: 10.1371/journal.pone.0210030 (PMC6329493; doi:10.1371/journal.pone.0210030)
Supplement: S1 File — (PDF) [file pone.0210030.s001.pdf]

PLEASE DO NOT WRITE ON THIS TEST FORM

Biology 1150 Pre-Course Survey

1. Please use a #**2 pencil** to mark your answer on the scantron sheet. Answer the questions honestly and to the best of your ability. No one is going to know it's you.

2. Write your **secret identifier** in the space that says "Name" on the scantron. This identifier consists of the first three letters of your mother's maiden name followed by the first three letters of the town in which you were born. For example, if your mother's maiden name is Smith, and you were born in Kalamazoo, your secret identifier is SMIKAL.

3. Write your **Lab Instructors' names** followed by your **Lecture instructor's name** in the space that says "Period" on the scantron.

Use the following information to answer the next 3 questions.
In fruit flies, the autosomal trait gray body (G) is dominant to black body (g)

1. Which of the following would be an example of a genotype?
a) GG
b) haploid
c) homologous chromosomes
d) black body

2. In a cross between a homozygous recessive individual and a heterozygous individual, what is the chance of getting a homozygous recessive offspring?
a) 100%
b) 75%
c) 50%
d) 25%
e) 0%

3. Which one or more of the following is(are) true of the gene that results in black body in fruit flies?
a) It is found on a chromosome.
b) It is made up of a series of DNA nucleotides.
c) It can be found in each fruit fly cell.
d) All of the above are true.
e) Only (b) and (c) are true.

Use the following information for the next 5 questions.
The castor oil plant (*Ricinus communis)* grows in temperate and tropical regions. Most varieties are green but some are red or purple. Many animals attempt to feed on the plant, but the plant produces a protein called ricin that is toxic to insects and other animals. The plant is not affected by the ricin because the protein is stored in its cells in an inactive form. Ricin becomes active however after it is ingested by animals. Once an animal ingests ricin, the protein binds to and inactivates the ribosomes in the animals cells.

4. During photosynthesis, light absorbed by the castor oil plant is used **directly** __.
   a. to convert carbon dioxide (CO_2) into oxygen (O_2)
   b. in cellular respiration
   c. to energize electrons in chlorophyll molecules
   d. to drive the chemiosmotic production of ATP in the chloroplast

5. When light from green (or red or purple) castor oil plants hits the retinas of our eyes, a nerve impulse transmits a message to our brain. Which of the following plays a part in this nerve impulse transmission?
   a) The formation of NADPH in the neurons.
   b) The opening of sodium channels and sodium ions entering neurons.
   c) The conversion of hypoxanthine to guanine outside the neurons.
   d) The action of the Michaelis-Menten pump along the neurons.

6. To study ricin, a scientist grows several of the green-leafed variety of castor oil plants in a lab. Under what conditions would you correctly predict the plants would grow fastest?
a) Under white light and in a cold room (5°C)
b) Under green light and in a warm room (30°C)
c) Under red light and in a cold room (5°C)
d) Under white light and in a warm room (30°C)

7. The scientist measures the carbon dioxide and oxygen in the air immediately surrounding the plants. Overnight (the temperature remains constant), you would correctly predict the air would_______.
a) decrease in carbon dioxide and increase in oxygen
b) increase in carbon dioxide and decrease in oxygen
c) increase in carbon dioxide and increase in oxygen
d) decrease in carbon dioxide and decrease in oxygen

8. A scientist predicts that the plants producing larger amounts of ricin will not photosynthesize as much as those that produce smaller amounts of ricin. The scientist collects data measuring the plant's rate of photosynthesis and ricin production during the day. The independent (manipulated) variable is _________ and the dependent (measured) variable is _________.
a) amount of ricin produced, oxygen consumed
b) oxygen consumed, amount of ricin produced
c) amount of ricin produced, carbon dioxide consumed
d) carbon dioxide consumed, amount of ricin produced

Use the information to answer the following 3 questions

If you watch late night infomercials on TV you may have seen this product: Kinoki Detox Foot Pads. Claimed to "pull" toxins from the body out through the soles of the feet while you sleep. The ad claims that for $19.95, one can enjoy better health, more energy, and quality of life as the Kinoki Foot Pads are "all-natural" and use "tree extracts" and "negative ions" to purge the body of "heavy metals, microscopic parasites, chemicals, and cellulite."

A researcher suspects the foot pads to be a scam, so designs the following experiment: She enlists 20 volunteers, 18-24 years old, who agree to strictly adhere to a standardized diet provided by the researcher for a two-week period. She assigns the volunteers to two groups: Group 1 wears the Kinoki foot pads every night as advertised for two weeks; Group 2 wears a simple cotton pad the same size and shape as the Kinoki foot pads. The researcher takes a blood sample from each volunteer at the start and end of the study, and looks for changes blood concentrations of the heavy metal mercury.

9. Which of the following represents the experimental group?
a) the volunteers who followed the standard diet
b) the volunteers who wore the Kinoki Foot Pads
c) the volunteers who wore the cotton foot pads
d) there was no experimental group because it was a correlational study

10. To best conduct an analysis of the potential effect of wearing Kinoki Foot Pads, which of the following should the researcher compare between her experimental and control groups?
a) the age of the volunteers
b) the treatment-whether the volunteers wore cotton pads of Kinoki pads
c) The concentration of mercury in the blood at the end of the trial
d) The change in mercury concentration from the first to the second blood samples.

11. At the conclusion of her study, the researcher summarized a portion of her analysis into the following figure. Based on the data presented, she correctly concludes that….

a) Kinoki foot pads reduced mercury concentrations in the blood more than the control did.

b) Kinoki foot pads did not reduce mercury concentrations in the blood.

c) Kinoki foot pads do not provide health benefits compared to the control.

d) Kinoki foot pads are more effective than diet in reducing mercury concentrations in the blood.

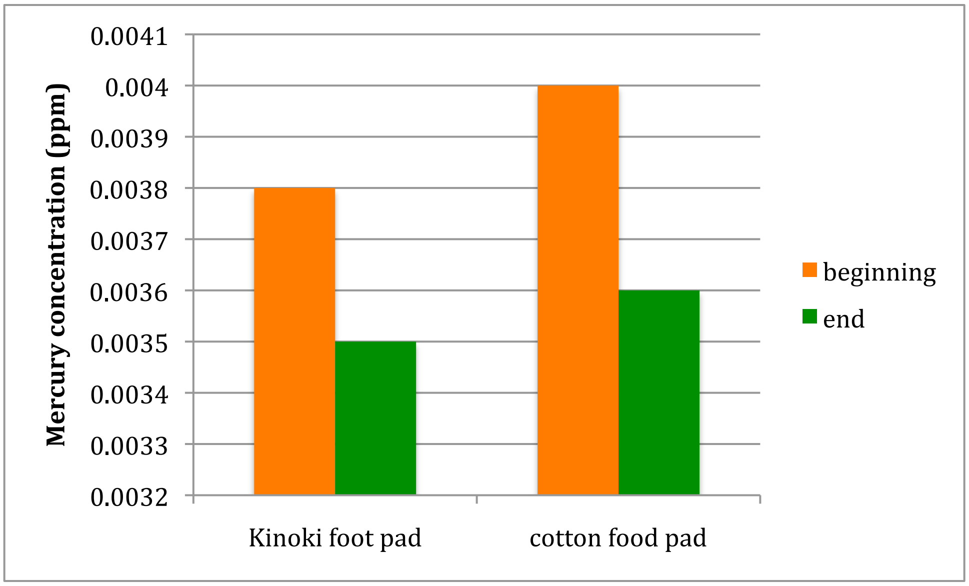

The remaining questions are not associated with a scenario.

12. The alpha female of a pack of 10 wolves in Yellowstone National Park gives birth to 4 cubs in the spring of the year. Over the summer, one of the cubs dies. All of the adult wolves survive. What is the per capita (or per individual) growth rate of the pack for this year?

a) 0. 3
b) 3
c) 4
d) 4/14

13. In aerobic cellular respiration in the mitochondrion of a cell, oxygen ____.

a) Combines with pyruvate to make glucose during glycolysis
b) Is added to NAD to make NADH in the Krebs or Citric Acid cycle
c) Accepts electrons from the electron transport system to make water
d) Passes through the ATP synthase to produce ATP

14. Which one of the following statements is **false**?
a) All chromosomes are the same size and shape.
b) For a particular gene, the alleles may be identical or different.
c) In diploid cells, chromosomes can be matched up into pairs.
d) One allele in a gene pair is found on one homolgous chromosome; the other allele is on the other homologous chromosome.

15. Some species of buttercup are resistant to herbicides (chemicals which kill plants). Which of the following hypotheses regarding herbicide resistance in the Tall Buttercup species is most likely to be correct?
a) To survive and reproduce, the Tall Buttercup had to mutate to become resistant
b) The plants that became resistant during their lifetime passed this trait to their offspring.
c) Buttercups with yellow flowers are more likely to be resistant
d) Those buttercups that were resistant produced more offspring and subsequent generations contained more individuals that were resistant.
e) The herbicide caused the plants to mutate in order to become resistant.

16. Fitness from an evolutionary viewpoint refers to…
a) physical strength
b) number of fertile offspring
c) number of mating opportunities
d) amount of resources (e.g., food, territory) possessed
e) maintaining a healthy Body Mass Index (BMI)

17. A family of leeches living in the freshwater PC pond decides to visit their distant marine worm relatives who live near Hilton Head in the Atlantic Ocean. The leeches are poikilotherms, and when they spend the day in the hot sun at the beach ________.
a) their metabolic rate increases compared to the rate at night
b) they consume more carbon dioxide in cellular respiration.
c) they produce more oxygen than they do in the PC pond.
d) they gain water by osmosis
e) they produce less ATP than they do in the PC pond.

18. The smallest (and youngest) leech rushes out into the saltwater of the ocean with its marine worm cousin. You correctly predict that this youngster…
a) enjoys the water and tells everyone else to hurry in
b) shrivels up due to the cold temperature of the water
c) shrivels up due to a loss of water
d) swells and bursts due to a gain of salt
e) can safely go in and out of the water all day.

19. The nucleotide makeup of a virus was analyzed. It was found to contain 30% G and 20% A. The content of C is ________ and T is ________ .
a) 30%, 20%
b) 20%, 30%
c) 30%, 30%
d) There is not enough information to calculate the content of C and T.

20. Certain antibiotics inhibit specific steps in the process that results in bacteria protein synthesis. An antibiotic that inhibits transcription will stop the synthesis of _________.
a) proteins only
b) mRNA only
c) DNA only
d) enzymes only
e) mRNA and proteins

21. Which of the following is true about proteins?
a) They are involved in active transport
b) Their function depends on their shape
c) They are involved in facilitated diffusion
d) A protein loses its function when it denatures
e) all of the above are true

22. In which of the following would you expect to find the highest number of mitochondria?
a) A skin cell
b) a fat storage cell
c) a muscle cell
d) a hair cell

23. Which of the following statements concerning a gradient is <u>not</u> correct.
a) a stronger temperature gradient will result in faster heat transfer
b) solute movement from a place of high concentration to a place of low concentration requires the input of ATP (energy).
c) In osmosis, water moves through a semipermiable membrane from the side of high water concentration to the side of low water concentration.
d) When a cold lizard perches on a hot rock, heat from the rock will move to the lizard by conduction.

24. I am interested in learning more about biology
a) Strongly Agree
b) Agree
c) Neither agree nor disagree
d) Disagree
e) Strongly Disagree

25. I think I am good at designing experiments
a) Strongly Agree
b) Agree
c) Neither agree nor disagree
d) Disagree
e) Strongly Disagree

26. I think Biology 105 will be a hard course
a) Strongly Agree
b) Agree
c) Neither agree nor disagree
d) Disagree
e) Strongly Disagree

27. I think I will be successful in this course
a) Strongly Agree
b) Agree
c) Neither agree nor disagree
d) Disagree
e) Strongly Disagree

28. I think I will learn more in Bio 105 if I work by myself instead of working with a group.
a) Strongly Agree
b) Agree
c) Neither agree nor disagree
d) Disagree
e) Strongly Disagree

29. I learn better when I am able to figure something out on my own, rather than when someone is telling me about it.
a) Strongly Agree
b) Agree
c) Neither agree nor disagree
d) Disagree
e) Strongly Disagree

30. I can identify with confidence the things that I need to study more and the things I need to study less.
a) Strongly Agree
b) Agree
c) Neither agree nor disagree
d) Disagree
e) Strongly Disagree

31. Memorizing information is the same as understanding it.
a) Strongly Agree
b) Agree
c) Neither agree nor disagree
d) Disagree
e) Strongly Disagree

32. Some of my skills or abilities cannot be improved with practice.
a) Strongly Agree
b) Agree
c) Neither agree nor disagree
d) Disagree
e) Strongly Disagree

33. I am more anxious than my peers when it comes to taking a test.
a) Strongly Agree
b) Agree
c) Neither agree nor disagree
d) Disagree
e) Strongly Disagree

34. At PC I intend to major in:
a) Humanities
b) Business
c) Science/Math/ Medicine
d) Education
e) Other

35. I am
a) Female.
b) Male.
c) Rather not say.

Thank you for completing this survey.

PLEASE DO NOT WRITE ON THIS TEST FORM - USE THE BACK OF YOUR ANSWER SHEET

Biology 1150 Post-Course Survey

1. Please use a #**2 pencil** to mark your answer on the scantron sheet. Answer the questions honestly and to the best of your ability. No one is going to know it's you.

2. Write your **secret identifier** in the space that says "Name" on the scantron. This identifier consists of the first three letters of your mother's maiden name followed by the first three letters of the town in which you were born. For example, if your mother's maiden name is Smith, and you were born in Kalamazoo, your secret identifier is SMIKAL.

3. Write the **date** in the space provided. Write your **Lab Instructors' names** followed by your **Lecture instructor's name** in the space that says "Period" on the scantron.

Use the following information to answer the next 3 questions.
In fruit flies, the autosomal trait gray body (G) is dominant to black body (g)

1. Which of the following would be an example of a genotype?
a) GG
b) haploid
c) homologous chromosomes
d) black body

2. In a cross between a homozygous recessive individual and a heterozygous individual, what is the chance of getting a homozygous recessive offspring?
a) 100%
b) 75%
c) 50%
d) 25%
e) 0%

3. Which one or more of the following is(are) true of the gene that results in black body in fruit flies?
a) It is found on a chromosome.
b) It is made up of a series of DNA nucleotides.
c) It can be found in each fruit fly cell.
d) All of the above are true.
e) Only (b) and (c) are true.

Use the following information for the next 5 questions.
The castor oil plant (*Ricinus communis)* grows in temperate and tropical regions. Most varieties are green but some are red or purple. Many animals attempt to feed on the plant, but the plant produces a protein called ricin that is toxic to insects and other animals. The plant is not affected by the ricin because the protein is stored in its cells in an inactive form. Ricin becomes active however after it is ingested by animals. Once an animal ingests ricin, the protein binds to and inactivates the ribosomes in the animals cells.

4. During photosynthesis, light absorbed by the castor oil plant is used **directly** __.
   a. to convert carbon dioxide (CO_2) into oxygen (O_2)
   b. in cellular respiration
   c. to energize electrons in chlorophyll molecules
   d. to drive the chemiosmotic production of ATP in the chloroplast

5. When light from green (or red or purple) castor oil plants hits the retinas of our eyes, a nerve impulse transmits a message to our brain. Which of the following plays a part in this nerve impulse transmission?
   a) The formation of NADPH in the neurons.
   b) The opening of sodium channels and sodium ions entering neurons.
   c) The conversion of hypoxanthine to guanine outside the neurons.
   d) The action of the Michaelis-Menten pump along the neurons.

6. To study ricin, a scientist grows several of the green-leafed variety of castor oil plants in a lab. Under what conditions would you correctly predict the plants would grow fastest?
a) Under white light and in a cold room (5°C)
b) Under green light and in a warm room (30°C)
c) Under red light and in a cold room (5°C)
d) Under white light and in a warm room (30°C)

7. The scientist measures the carbon dioxide and oxygen in the air immediately surrounding the plants. Overnight (the temperature remains constant), you would correctly predict the air would_______.
a) decrease in carbon dioxide and increase in oxygen
b) increase in carbon dioxide and decrease in oxygen
c) increase in carbon dioxide and increase in oxygen
d) decrease in carbon dioxide and decrease in oxygen

8. A scientist predicts that the plants producing larger amounts of ricin will not photosynthesize as much as those that produce smaller amounts of ricin. The scientist collects data measuring the plant's rate of photosynthesis and ricin production during the day. The independent (manipulated) variable is _________ and the dependent (measured) variable is _________.
a) amount of ricin produced, oxygen consumed
b) oxygen consumed, amount of ricin produced
c) amount of ricin produced, carbon dioxide consumed
d) carbon dioxide consumed, amount of ricin produced

Use the information to answer the following 3 questions

If you watch late night infomercials on TV you may have seen this product: Kinoki Detox Foot Pads. Claimed to "pull" toxins from the body out through the soles of the feet while you sleep. The ad claims that for $19.95, one can enjoy better health, more energy, and quality of life as the Kinoki Foot Pads are "all-natural" and use "tree extracts" and "negative ions" to purge the body of "heavy metals, microscopic parasites, chemicals, and cellulite."

A researcher suspects the foot pads to be a scam, so designs the following experiment: She enlists 20 volunteers, 18-24 years old, who agree to strictly adhere to a standardized diet provided by the researcher for a two-week period. She assigns the volunteers to two groups: Group 1 wears the Kinoki foot pads every night as advertised for two weeks; Group 2 wears a simple cotton pad the same size and shape as the Kinoki foot pads. The researcher takes a blood sample from each volunteer at the start and end of the study, and looks for changes blood concentrations of the heavy metal mercury.

9. Which of the following represents the experimental group?
  a) the volunteers who followed the standard diet
  b) the volunteers who wore the Kinoki Foot Pads
  c) the volunteers who wore the cotton foot pads
  d) there was no experimental group because it was a correlational study

10. To best conduct an analysis of the potential effect of wearing Kinoki Foot Pads, which of the following should the researcher compare between her experimental and control groups?
  a) the age of the volunteers
  b) the treatment-whether the volunteers wore cotton pads of Kinoki pads
  c) The concentration of mercury in the blood at the end of the trial
  d) The change in mercury concentration from the first to the second blood samples.

11. At the conclusion of her study, the researcher summarized a portion of her analysis into the following figure. Based on the data presented, she correctly concludes that….

a) Kinoki foot pads reduced mercury concentrations in the blood more than the control did.

b) Kinoki foot pads did not reduce mercury concentrations in the blood.

c) Kinoki foot pads do not provide health benefits compared to the control.

d) Kinoki foot pads are more effective than diet in reducing mercury concentrations in the blood.

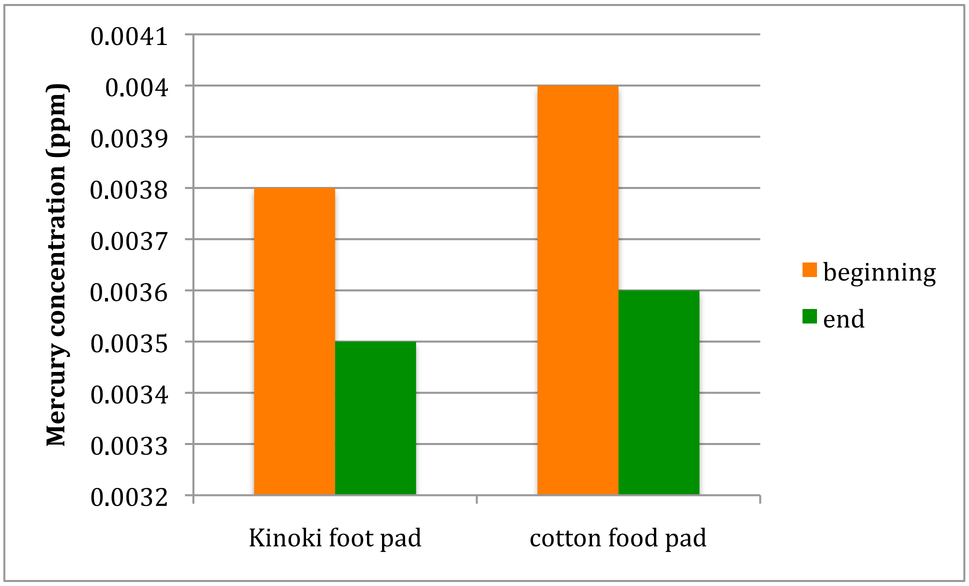

The remaining questions are not associated with a scenario.

12. The alpha female of a pack of 10 wolves in Yellowstone National Park gives birth to 4 cubs in the spring of the year. Over the summer, one of the cubs dies. All of the adult wolves survive. What is the per capita (or per individual) growth rate of the pack for this year?

a) 0. 3
b) 3
c) 4
d) 4/14

13. In aerobic cellular respiration in the mitochondrion of a cell, oxygen ____.

a) Combines with pyruvate to make glucose during glycolysis
b) Is added to NAD to make NADH in the Krebs or Citric Acid cycle
c) Accepts electrons from the electron transport system to make water
d) Passes through the ATP synthase to produce ATP

14. Which one of the following statements is **false**?
a) All chromosomes are the same size and shape.
b) For a particular gene, the alleles may be identical or different.
c) In diploid cells, chromosomes can be matched up into pairs.
d) One allele in a gene pair is found on one homolgous chromosome; the other allele is on the other homologous chromosome.

15. Some species of buttercup are resistant to herbicides (chemicals which kill plants). Which of the following hypotheses regarding herbicide resistance in the Tall Buttercup species is most likely to be correct?
a) To survive and reproduce, the Tall Buttercup had to mutate to become resistant
b) The plants that became resistant during their lifetime passed this trait to their offspring.
c) Buttercups with yellow flowers are more likely to be resistant
d) Those buttercups that were resistant produced more offspring and subsequent generations contained more individuals that were resistant.
e) The herbicide caused the plants to mutate in order to become resistant.

16. Fitness from an evolutionary viewpoint refers to…
a) physical strength
b) number of fertile offspring
c) number of mating opportunities
d) amount of resources (e.g., food, territory) possessed
e) maintaining a healthy Body Mass Index (BMI)

17. A family of leeches living in the freshwater PC pond decides to visit their distant marine worm relatives who live near Hilton Head in the Atlantic Ocean. The leeches are poikilotherms, and when they spend the day in the hot sun at the beach ________.
a) their metabolic rate increases compared to the rate at night
b) they consume more carbon dioxide in cellular respiration.
c) they produce more oxygen than they do in the PC pond.
d) they gain water by osmosis
e) they produce less ATP than they do in the PC pond.

18. The smallest (and youngest) leech rushes out into the saltwater of the ocean with its marine worm cousin. You correctly predict that this youngster…
a) enjoys the water and tells everyone else to hurry in
b) shrivels up due to the cold temperature of the water
c) shrivels up due to a loss of water
d) swells and bursts due to a gain of salt
e) can safely go in and out of the water all day.

19. The nucleotide makeup of a virus was analyzed. It was found to contain 30% G and 20% A. The content of C is ________ and T is ________ .
a) 30%, 20%
b) 20%, 30%
c) 30%, 30%
d) There is not enough information to calculate the content of C and T.

20. Certain antibiotics inhibit specific steps in the process that results in bacteria protein synthesis. An antibiotic that inhibits transcription will stop the synthesis of _________.
a) proteins only
b) mRNA only
c) DNA only
d) enzymes only
e) mRNA and proteins

21. Which of the following is true about proteins?
a) They are involved in active transport
b) Their function depends on their shape
c) They are involved in facilitated diffusion
d) A protein loses its function when it denatures
e) all of the above are true

22. In which of the following would you expect to find the highest number of mitochondria?
a) A skin cell
b) a fat storage cell
c) a muscle cell
d) a hair cell

23. Which of the following statements concerning a gradient is <u>not</u> correct.
a) a stronger temperature gradient will result in faster heat transfer
b) solute movement from a place of high concentration to a place of low concentration requires the input of ATP (energy).
c) In osmosis, water moves through a semipermiable membrane from the side of high water concentration to the side of low water concentration.
d) When a cold lizard perches on a hot rock, heat from the rock will move to the lizard by conduction.

24. I am interested in learning more about biology
a) Strongly Agree
b) Agree
c) Neither agree nor disagree
d) Disagree
e) Strongly Disagree

25. I think I am good at designing experiments
a) Strongly Agree
b) Agree
c) Neither agree nor disagree
d) Disagree
e) Strongly Disagree

26. I think Biology 105 was a hard course
a) Strongly Agree
b) Agree
c) Neither agree nor disagree
d) Disagree
e) Strongly Disagree

27. I think I was successful in this course
a) Strongly Agree
b) Agree
c) Neither agree nor disagree
d) Disagree
e) Strongly Disagree

28. I think I would have learned more in Bio 105 if I worked by myself instead of working with a group.
a) Strongly Agree
b) Agree
c) Neither agree nor disagree
d) Disagree
e) Strongly Disagree

29. I learn better when I am able to figure something out on my own, rather than when someone is telling me about it.
a) Strongly Agree
b) Agree
c) Neither agree nor disagree
d) Disagree
e) Strongly Disagree

30. I can identify with confidence the things that I need to study more and the things I need to study less.
a) Strongly Agree
b) Agree
c) Neither agree nor disagree
d) Disagree
e) Strongly Disagree

31. Memorizing information is the same as understanding it.
a) Strongly Agree
b) Agree
c) Neither agree nor disagree
d) Disagree
e) Strongly Disagree

32. Some of my skills or abilities cannot be improved with practice.
a) Strongly Agree
b) Agree
c) Neither agree nor disagree
d) Disagree
e) Strongly Disagree

33. I am more anxious than my peers when it comes to taking a test.
a) Strongly Agree
b) Agree
c) Neither agree nor disagree
d) Disagree
e) Strongly Disagree

34. At PC I intend to major in:
a) Humanities
b) Business
c) Science/Math/ Medicine
d) Education
e) Other

35. I do not plan to take another biology course at PC.
a) Strongly Agree
b) Agree
c) Neither agree nor disagree
d) Disagree
e) Strongly Disagree

36. I am curious about biological issues that relate to everyday life.
a) Strongly Agree
b) Agree
c) Neither agree nor disagree
d) Disagree
e) Strongly Disagree

Thank you for completing this survey.
