# Supplementary material for: Knowing is half the battle: Assessments of both student perception and performance are necessary to successfully evaluate curricular transformation
Source: PLoS One. 2019 Jan 11;14(1):e0210030. doi: 10.1371/journal.pone.0210030 (PMC6329493; doi:10.1371/journal.pone.0210030)
Supplement: S2 File — (PDF) [file pone.0210030.s002.pdf]

Supporting Information: Additional SALG Analysis

To examine the changes in how students perceived specific aspects of the course, we used the following items from our SALG instrument:

3.1) As a result of this class, what gains did you make in your enthusiasm for biology? (S1 Fig)
3.3) As a result of this class, what gains did you make in your interest in taking more classes in biology? (S2 Fig)
5.1) How much did the instructional approach taken in this class help your learning? (S3 Fig)
6.4) How much did participating in group work help your learning? (S4 Fig)
7.2.1) How much did having clicker questions help your learning? (S5 Fig)
7.3) How much did having online quizzes help your learning? (S6 Fig)
9.1) How much did the explanation of how the course content, homework and exams related to each other help your learning? (S7 Fig)
10.8) How much help was working with a Peer Mentor during lab or in the Learning Resource Center? (S8 Fig)

For each question, we compared across years the frequencies of student responses for the different ranks of no gain, little gain, moderate gain, good gain, and great gain using chi-squared tests. To examine if STEM majors vs. non-STEM majors view active learning pedagogies differently, we used Fisher's exact tests to compare how the different student groups responded Question 5.1. Note that we did not gather information on student majors in 2013.

As a result of this class, what gains did you make in your enthusiasm for biology?

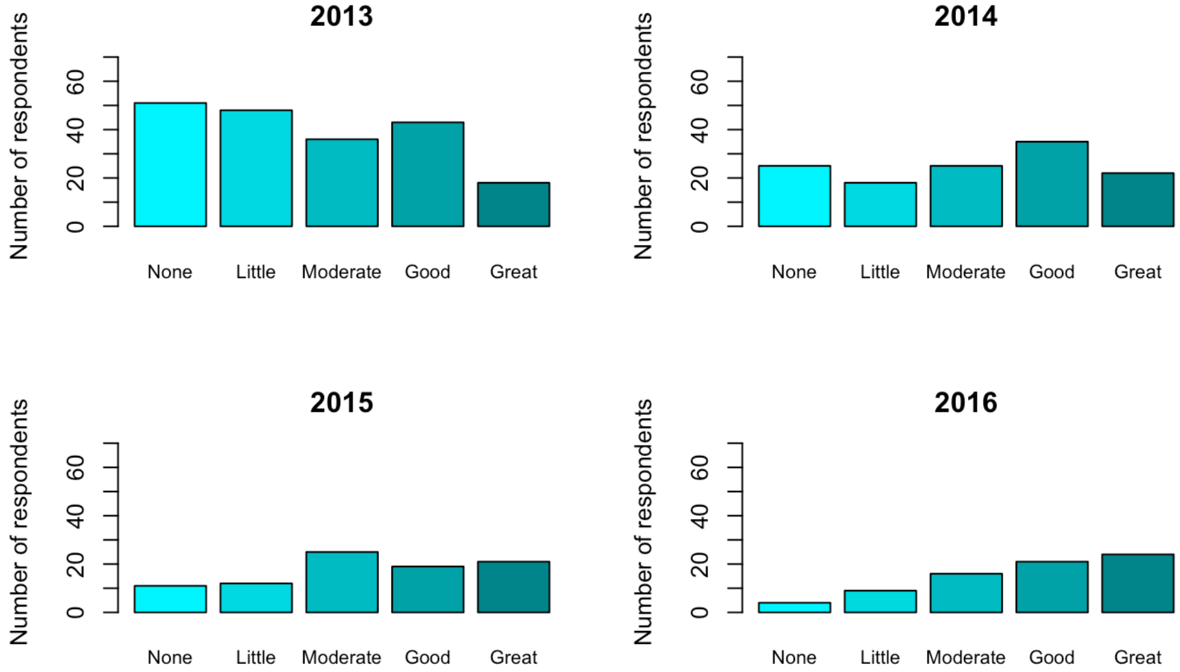

S1 Fig. Student responses shift over time, reducing the frequency of low rankings (None, Little) relative to high rankings (Good, Great). Students increasingly recognize the class as responsible for higher gains in their enthusiasm for biology over four years ($p<0.0001$).

As a result of this class, what gains did you make in your interest in taking more classes in biology?

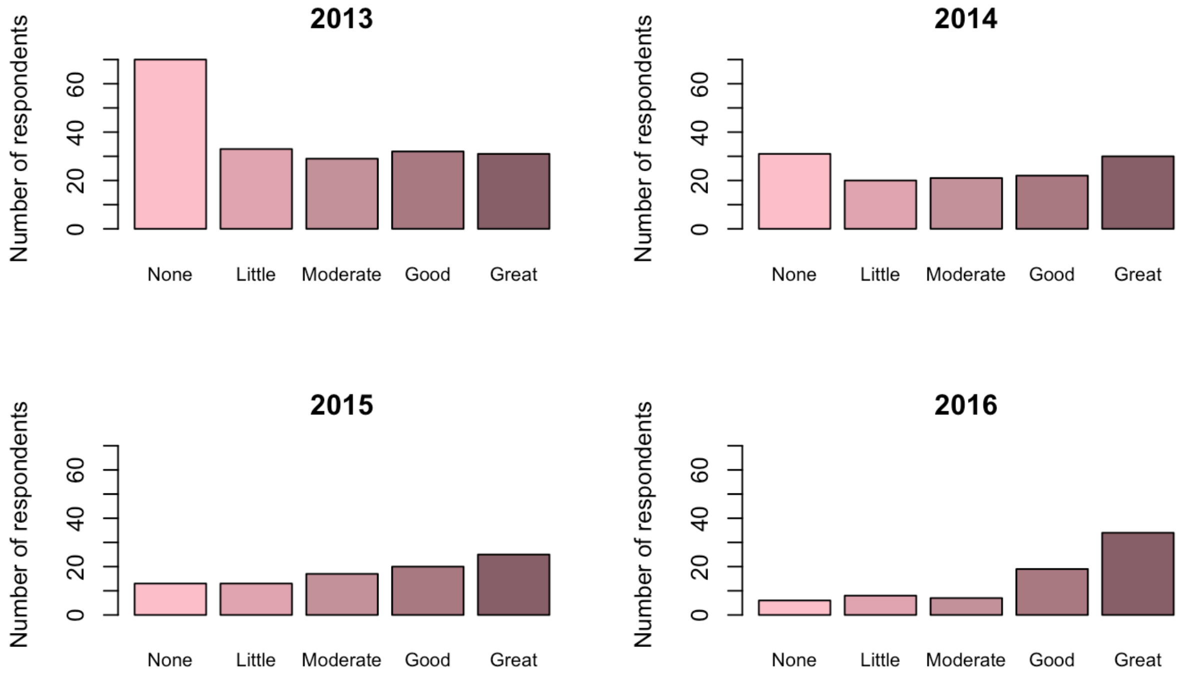

S2 Fig. Student responses shift over time, reducing the frequency of low rankings (None, Little) relative to high rankings (Good, Great). Over four years, students increasingly recognize the class as responsible for higher gains in their desire to take more biology courses ($p<0.0001$).

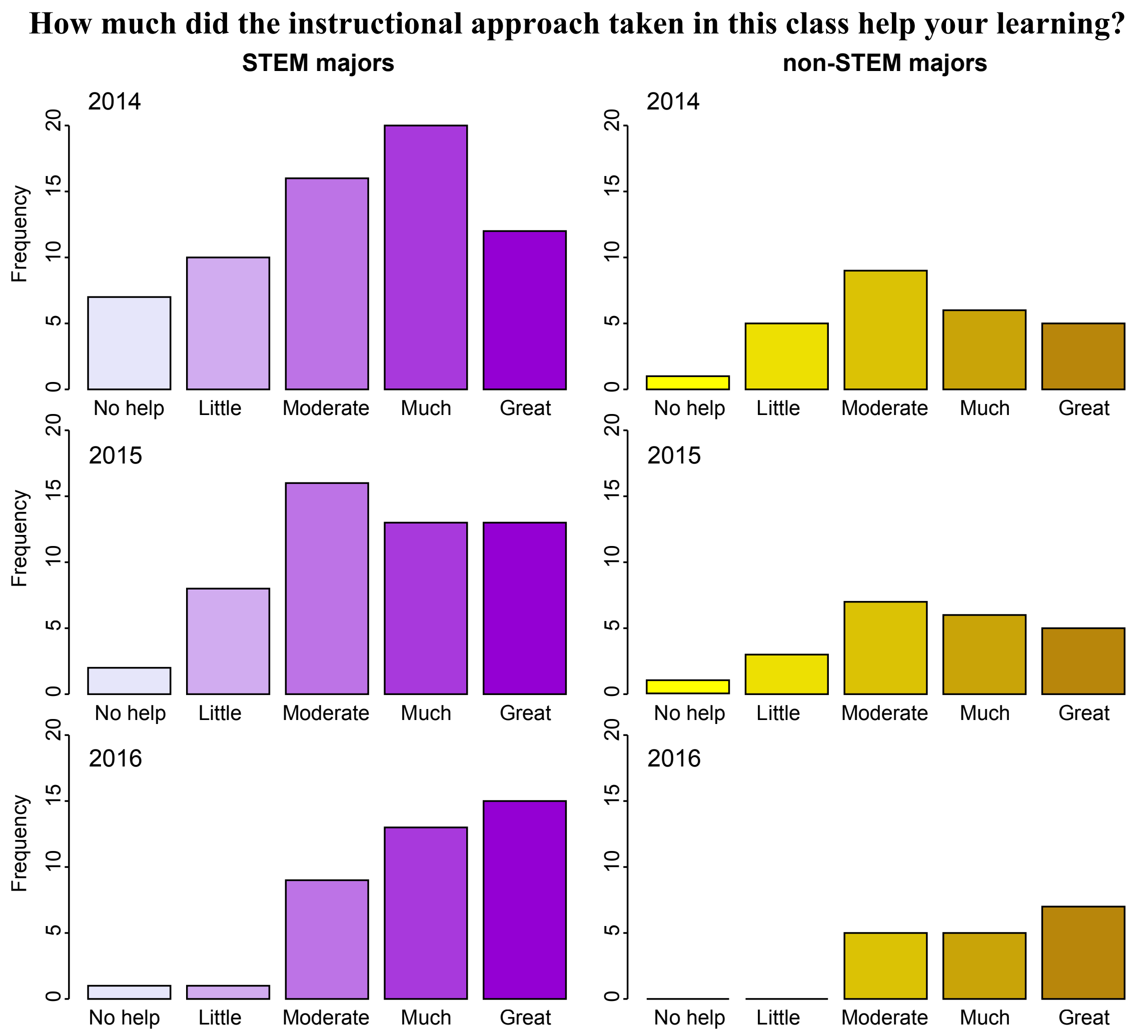

S3 Fig. Student major did not influence their perceptions of how the instructional approach taken in the class helped their learning in 2014 (p=0.09), 2015 (p=0. 20), and 2016 (p=0.9677).

How much did participating in group work help your learning?

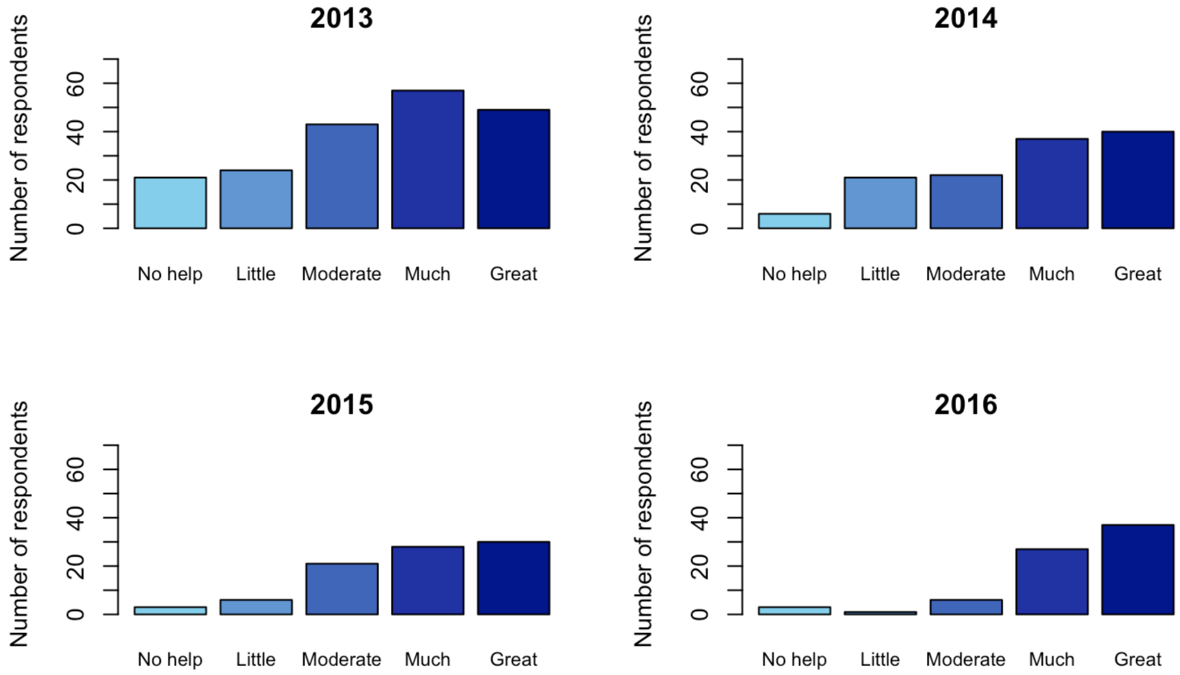

S4 Fig. Student responses shift over time, reducing the frequency of low rankings (None, Little) relative to high rankings (Good, Great). Over four years, students increasingly report that participating in group work during class as responsible for large gains in their learning (p=0.0002).

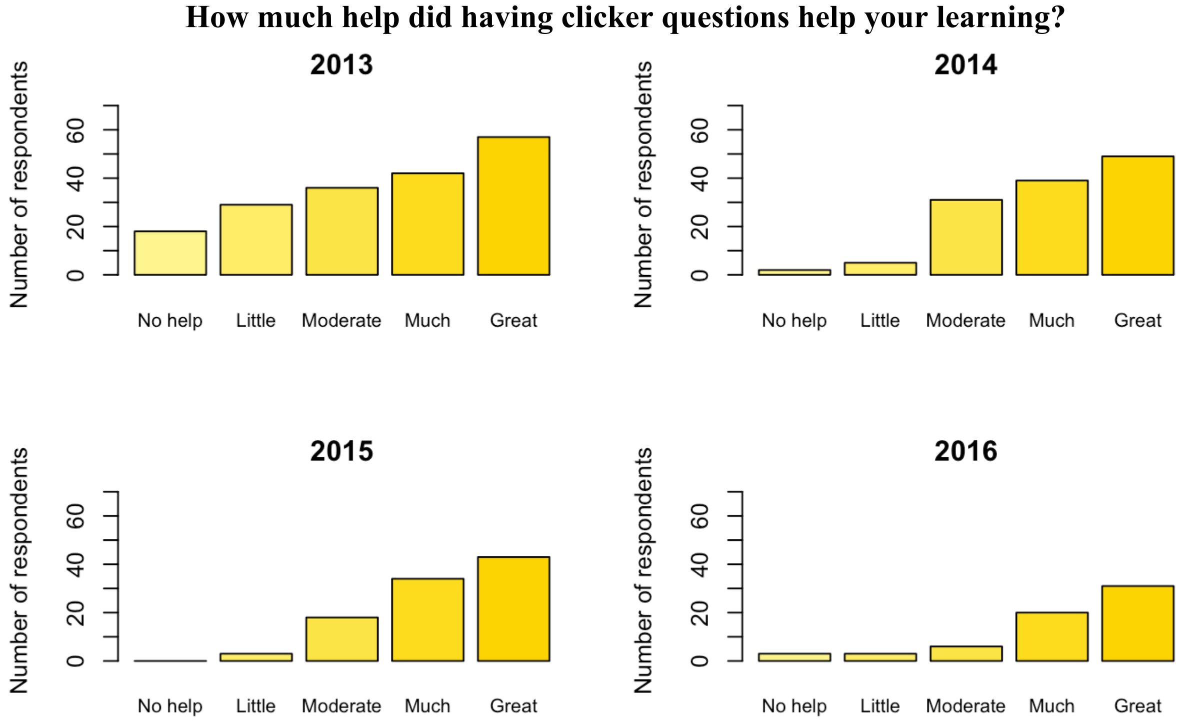

S5 Fig. Over time, students reduced their assessment of clicker questions as being of No or Little Help, and increased the proportion of students who viewed clicker questions as being Much or Great Help (*p*=0.0005).

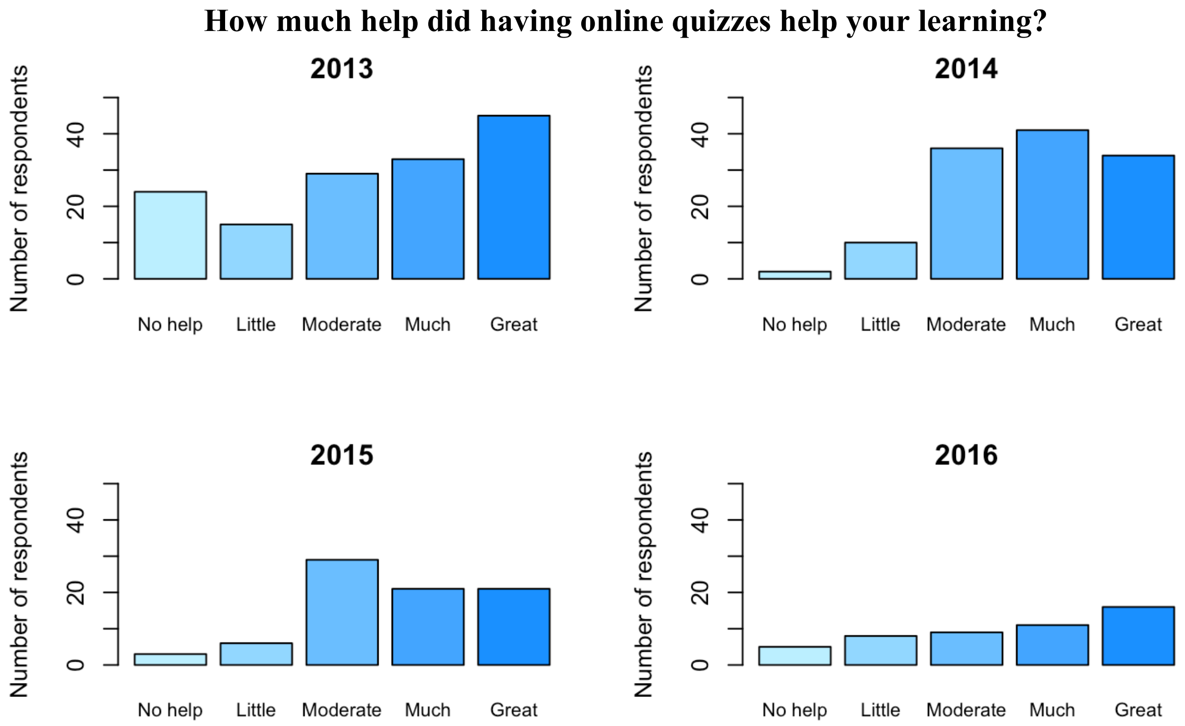

S6 Fig. Students varied in how helpful they thought online quizzes were for their learning, though in 2014 and 2015, most students thought they were of at least Moderate Help (p=0.0007).

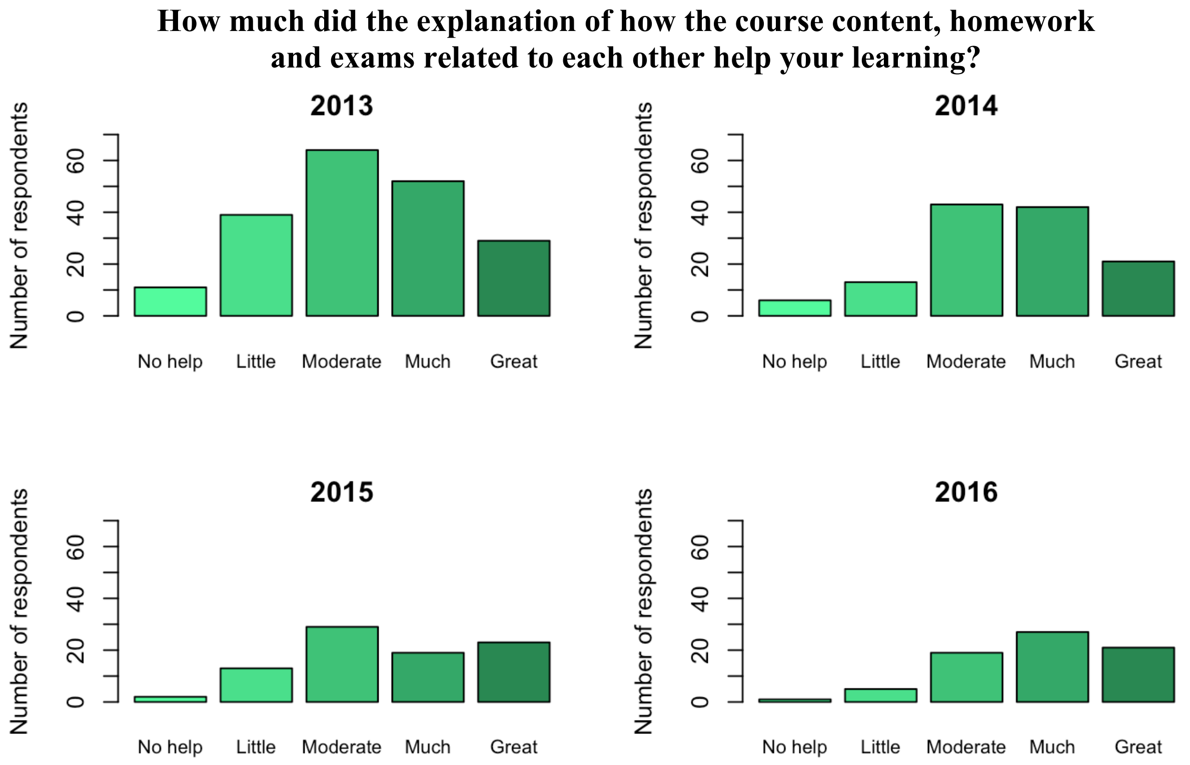

S7 Fig. Students shifted in how helpful they thought was the explanation of the alignment of course components–over time, fewer students saw this explanation as No or Little help (p=0.014).

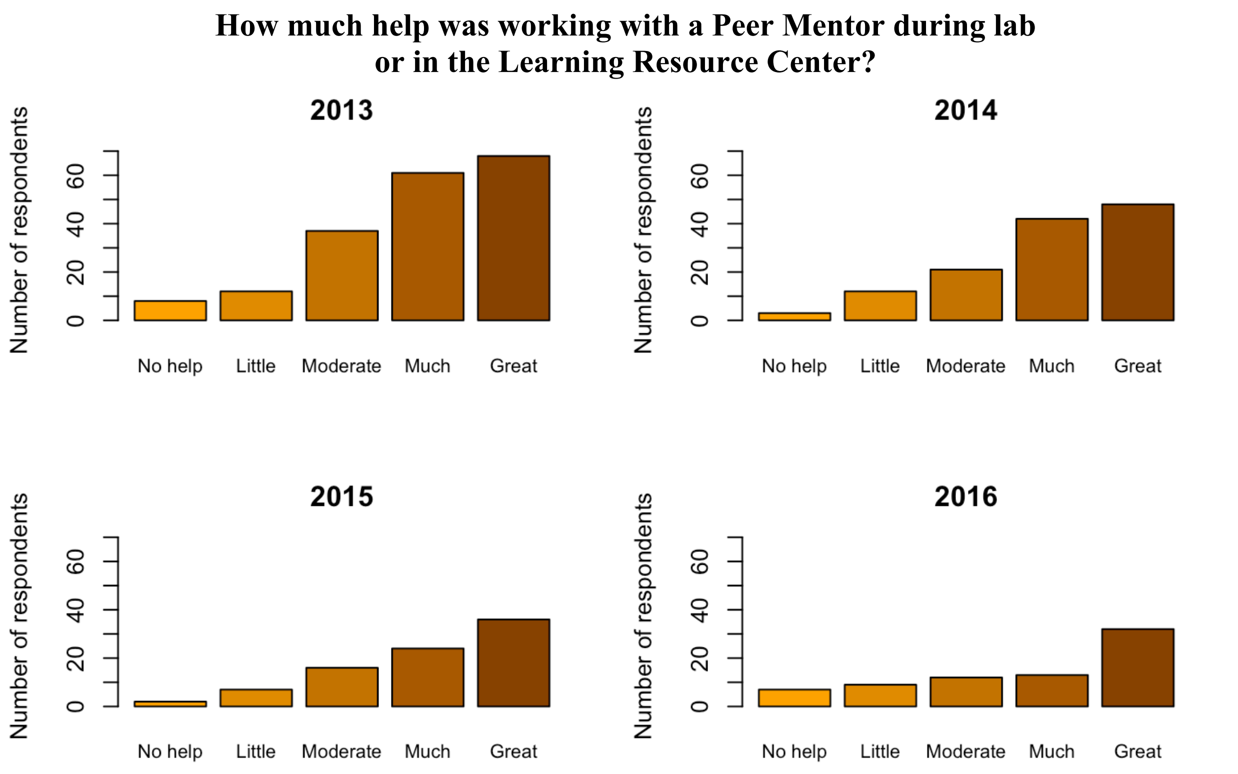

S8 Fig. Student responses across categories did not vary in relative frequency during the study (*p*=0.23). Working with a Peer Mentor was consistently and predominantly seen as Much Help or Great Help.
